# A 15-Year-Old Boy with Anterior Chest Pain, Progressive Dyspnea, and Subcutaneous Emphysema of the Neck

**DOI:** 10.1155/2009/496890

**Published:** 2009-02-01

**Authors:** Nicola Scichilone, Maria Buttacavoli, Gaetana Camarda, Margherita Marchese, Maria Bellia, Mario Spatafora

**Affiliations:** ^1^Dipartimento di Medicina, Pneumologia, Fisiologia e Nutrizione Umana (DIMPEFINU), Sezione di Pneumologia e Medicina, University of Palermo, 90146 Palermo, Italy; ^2^Dipartimento di Biotecnologie e Medicina Legale, Sezione di Scienze Radiologiche, University of Palermo, 90127 Palermo, Italy

## Abstract

We describe the case of an adolescent who was admitted to the hospital because of sudden occurrence of chest pain, dyspnea and subcutaneous emphysema. On admission, physical examination revealed subcutaneous crepitations in the superior part of the rib cage, and auscultation of the chest showed widespread wheezing. The radiological assessment confirmed the diagnosis of pneumomediastinum and pneumothorax. A follow-up CT scan performed one week after the admission showed almost complete resolution of the radiological alterations. At the following visits, the patient was asymptomatic, but reported to have suffered from frequent episodes of rhinorrea, sneezing, nasal blockage, and sometimes, chest tightness, especially during exposure to pets and/or windy weather. Skin prick testing showed sensitivities to dermatophagoides pteronyssinus and farinae, grass pollen and dog dander. Spirometry documented significant improvement in lung function after short-acting bronchodilator, allowing for the diagnosis of asthma to be made. Although pneumomediastinum may be a complication of various respiratory diseases, including asthma, it has never been reported as the first presentation of underlying bronchial asthma. Herein, the physiopathological mechanisms, the diagnostic procedures and treatment of pneumomediastinum in asthma are discussed. We suggest that the diagnosis of asthma should be considered in the differential diagnosis of pneumomediastinum in adolescence.

## 1. Case Report

A 15-year-old boy was admitted to the emergency room with anterior chest pain, nonproductive cough, progressive dyspnea, and subcutaneous emphysema of the neck. All symptoms had occurred suddenly, although the night before and the morning of the acute respiratory distress he had experienced heavy breathing, for which he had arbitrarily taken oral corticosteroids. He denied accidental trauma of the chest, surgical maneuvers, or acute infections prior to the respiratory event. Also, chronic respiratory or nonrespiratory diseases were not referred. He was neither a smoker nor a recreational drug user.

On admission, he was eupnoic at rest, respiratory rate was 18/min, oxygen saturation was 98% on room air, pulse rate was regular, blood pressure was 110/60 mm Hg, and body temperature was 36.5°C. Physical examination revealed extensive subcutaneous crepitations along the neck region and the superior part of the rib cage. Auscultation of the chest showed widespread wheezing. No other abnormalities of the chest and the abdomen were detected. Laboratory studies revealed Hb 15.7 gr/dL, and WBC counts11.2 × 10^9^/l (neutrophils 92.7%, lymphocytes 6.7%, and eosinophils 0.1%). Renal and liver function tests were within the normal range. An ECG indicated a regular sinus rhythm and normal voltage.

**Figure 1:**
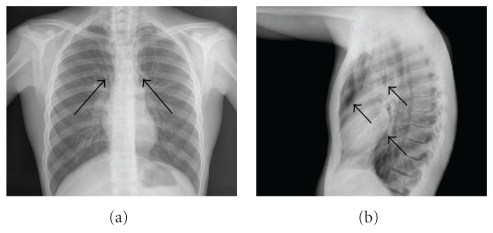
(a) Posteroanterior chest radiograph that shows the mediastinal reflections of the pleura separated from the pericardium by a lucent band of air representing pneumomediastinum (arrows). (b) Lateral chest radiograph that shows the outer border of the ascending and descending thoracic aorta, which are underlined by mediastinal free air collection (arrows).

The patient was referred for posteroanterior and lateral chest X-ray (Figures 1(a) and 1(b)), which demonstrated linear streaks of air in the mediastinum extending into the upper parts of the lung, more evident in the lateral projection. The radiological signs were suggestive of pneumomediastinum. The radiological signs of pneumomediastinum are multiple and include radiolucent linear streaks of air in the mediastinum, often extending into the neck, air surrounding the mediastinal structures; the presence of subcutaneous emphysema of soft tissues is often described. The lateral view increases the sensitivity in detecting signs of pneumomediastinum, in that, it may reveal radiolucent bands in the retrosternal areas, such as in our patient. For further evaluation, he underwent thoracic high-resolution computed tomography (HRCT), where air was demonstrated around the esophagus, trachea, ascending aorta, peribronchial, and perivascular connective tissue; partial pneumothorax on both sides was detected; this was more prominent at the level of the left apex of the lung. Finally, diffuse subcutaneous emphysema was present. No bullae or cystic malformations were demonstrated in the lungs.

A bronchoscopy revealed viscous exudate occluding the segmental bronchi bilaterally. No other abnormalities of the bronchial tree, such as fistulae, were detected. The exudate was aspirated, and bronchial lavage with saline solution was performed. The patient was treated with systemic and inhaled corticosteroids plus inhaled long-acting bronchodilators; analgesic treatment was administered when necessary. A follow-up CT scan performed one week after the admission showed almost complete resolution of the radiological alterations. The patient was discharged with no medication and was strongly advised to maintain a resting lifestyle. He was invited to return to the outpatient clinic for follow-up investigations.

At the follow-up visits, he was asymptomatic, and physical and radiological examinations were normal. He had no history of asthma or other chronic respiratory diseases. However, his mother reported an episode of acute dyspnea when he was 4 years old. In the year before the event, he had suffered from frequent episodes of rhinorrea, sneezing, nasal blockage, and sometimes chest tightness, especially during exposure to pets and/or windy weather. Skin prick testing was performed, revealing sensitivities to dermatophagoides pteronyssinus and farinae, grass pollen, and dog dander. Total IgE were 146 IU/ml; specific IgE for dermatophagoides pteronyssinus and farinae was 0.470 kU/l and 13.9 kU/l, respectively; IgE values for dog dander were 11.3 kU/l, and for grass pollen 4.9 kU/l (normal range for specific IgE < 0.10 kU/l). Spirometry documented significant improvement in lung function after short-acting bronchodilator (15% from baseline FEV_1_), which allowed for the diagnosis of asthma to be made.

## 2. Discussion

The diagnosis of asthma is rarely a challenge for physicians; usually, a suspicion arising from the clinical manifestations of the disease is supported by the results of the functional pulmonary tests showing signs of reversible bronchial obstruction, which render the diagnosis an uncomplicated task. In some circumstances, however, the diagnostic process may be delayed or influenced by unusual presentation of asthma. This occurs especially in elderly patients, in whom multiple respiratory symptoms may be confusing or in subjects with noncharacteristic symptoms, in whom the single respiratory symptom may be misleading.

In our case, pneumomediastinum and pneumotorax represented the first presentation of allergic asthma. Atypical presentations of asthma have been associated with cough as the only respiratory symptom (cough-variant asthma) [1]; urticaria [2] or anaphylactic shock [3] is known as first appearance of an underlying asthma. Pneumomediastinum has been reported as an unusual, rare complication of bronchial asthma [4, 5]. Reports on this topic are scarce and all refer to spontaneous pneumomediastinum and subcutaneous emphysema following an acute attack of asthma. To our knowledge, this is the first clinical report of pneumomediastinum as initial sign of undiagnosed asthma. On the basis of the documented sensitization to grass pollen in our patient, and given that the episode occurred in September when the pollen concentration is still elevated in Southern Italy, the role of the allergic component as the precipitating factor cannot be excluded.

Pneumotorax may occur if the mediastinal pressure rises abruptly. The air leakage is usually the result of alveolar wall rupture secondary to high intra-alveolar pressures. Histological studies [6] have shown that air dissects into the connective tissue, resulting in interstitial emphysema. Because of a pressure gradient between the periphery of the lung and the hilus, air tracks along the vascular sheath into the hilum, thus reaching the mediastinum and moving to the neck and the subcutaneous space. Sometimes, air may also decompress into the pericardium or retroperitoneal tissues. Causes of increased alveolar pressure include barotrauma in patients receiving mechanical ventilation, deep inspiratory maneuvers such as during strenuous exercise or diabetic ketoacidosis, extreme respiratory efforts such as violent cough or prolonged Valsalva manoeuver, and obstructed expiratory flow with overinflation. The clinical description of our patient leaves no doubt on the obstructive origin of the pneumomediastinum. Asthma has been described in different studies as a predisposing factor for the development of spontaneous pneumomediastinum in up to 50% of cases [7–10]. In the retrospective study of Macia et al. [11] 9 out of 41 patients (22%) had a recent of remote history of asthma, and a severe attack precipitating the pneumomediastinum was demonstrated in only one out of four asthmatics. Nonrespiratory causes of pneumomediastinum are represented by disruption of the esophagus, penetrating thoracic injuries, or mediastinal infection with gas-forming bacteria. The clinical history and the absence of symptoms suggestive of the above-described conditions in our patient allowed ruling out nonrespiratory causes of the pneumomediastinum.

The incidence of spontaneous pneumomediastinum is difficult to establish due to the paucity of studies [11, 12]. The clinical diagnosis is based on the symptom triad of dyspnea, nonspecific chest pain, and subcutaneous emphysema, which was evident in our case. However, symptoms are nonspecific, and sometimes the patient may be asymptomatic. The only patognomonic sign (Hamman's sign), [13] characterized by a crunching or bubbling sound that is synchronous with the heart beat, is rarely noticed by the patient or the physician. In a revision of 41 cases with pneumomediastinum, Hamman's sign was detected in only 12% of patients. This contributes to underestimate the real incidence of spontaneous pneumomediastinum. Our patient only referred progressive dyspnea and chest pain; no other respiratory and nonrespiratory symptoms were present; Hamman's sign was not detected at the time of presentation.

The computed tomography (CT) scanning is considered the gold standard of imaging tests because it is able to detect even small amounts of mediastinal air. The CT scan has the advantage over the chest radiographs of offering superior contrast resolution and lack of superimposition; therefore, the sensitivity of the methodology is greater, enabling the false negative cases to be reduced. In our case, the CT scans allowed to detect the initial condition and to follow the clinical and radiological evolutions.

In addition to the evaluation and treatment of the underlying condition, the typical management of pneumomediastinum consists of rest, oxygen therapy, and analgesia. Our patient was treated with bed rest and analgesics; oxygen and cough sedatives were not required. After the discharge, he was strongly invited to follow a rigid resting lifestyle, and asthma was treated according to the most recent guidelines.

In conclusion, pneumomediastinum with pneumothorax and subcutaneous emphysema may represent a very unusual presentation of underlying allergic asthma. Pneumomediastinum is a benign condition, which occurs spontaneously or as a complication of an underlying respiratory disease, such as during an asthma attack. This is, to our knowledge, the first report of pneumomediastinum as asthma symptom, rather than asthma complication. In the diagnostic evaluation of the causes of pneumomediastinum in young subjects, the presence of underlying asthma should always be considered.
